# Measuring clinical management by physicians and nurses in European hospitals: development and validation of two scales

**DOI:** 10.1093/intqhc/mzu014

**Published:** 2014-03-09

**Authors:** Thomas Plochg, Onyebuchi A. Arah, Daan Botje, Caroline A. Thompson, Niek S. Klazinga, Cordula Wagner, Russell Mannion, Kiki Lombarts, on behalf of the DUQuE Project Consortium, NS Klazinga, DS Kringos, MJMH Lombarts, T Plochg, MA Lopez, M Secanell, R Sunol, P Vallejo, P Bartels, S Kristensen, P Michel, F Saillour-Glenisson, F Vlcek, M Car, S Jones, E Klaus, S Bottaro, P Garel, M Saluvan, C Bruneau, A Depaigne-Loth, C Shaw, A Hammer, O Ommen, H Pfaff, O Groene, D Botje, C Wagner, H Kutaj-Wasikowska, B Kutryba, A Escoval, A Lívio, M Eiras, M Franca, I Leite, F Almeman, H Kus, K Ozturk, R Mannion, OA Arah, M DerSarkissian, CA Thompson, A Wang, A Thompson

**Affiliations:** 1 Department of Public Health, Academic Medical Center, University of Amsterdam, Amsterdam, The Netherlands; 2 Department of Epidemiology, Fielding School of Public Health, University of California, Los Angeles (UCLA), Los Angeles, CA, USA; 3 UCLA Center for Health Policy Research, Los Angeles, USA; 4 NIVEL, Netherlands Institute for Health Services Research, Utrecht, The Netherlands; 5 Palo Alto Medical Foundation Research Institute, Palo Alto, CA, USA; 6 Department of Public and Occupational Health, EMGO Institute for Health and Care Research, VU University Medical Center, Amsterdam, The Netherlands; 7 Health Services Management Centre, University of Birmingham, Birmingham B15 2RT, UK; 8 Professional Performance Research Group, Center for Evidence-Based Education, Academic Medical Center, University of Amsterdam, Amsterdam, The Netherlands

**Keywords:** clinical management, professional involvement, quality systems, hospital management

## Abstract

**Objective:**

Clinical management is hypothesized to be critical for hospital management and hospital performance. The aims of this study were to develop and validate professional involvement scales for measuring the level of clinical management by physicians and nurses in European hospitals.

**Design:**

Testing of validity and reliability of scales derived from a questionnaire of 21 items was developed on the basis of a previous study and expert opinion and administered in a cross-sectional seven-country research project ‘Deepening our Understanding of Quality improvement in Europe’ (DUQuE).

**Setting and Participants:**

A sample of 3386 leading physicians and nurses working in 188 hospitals located in Czech Republic, France, Germany, Poland, Portugal, Spain and Turkey.

**Main Outcome Measures:**

Validity and reliability of professional involvement scales and subscales.

**Results:**

Psychometric analysis yielded four subscales for leading physicians: (i) Administration and budgeting, (ii) Managing medical practice, (iii) Strategic management and (iv) Managing nursing practice. Only the first three factors applied well to the nurses. Cronbach's alpha for internal consistency ranged from 0.74 to 0.86 for the physicians, and from 0.61 to 0.81 for the nurses. Except for the 0.74 correlation between ‘Administration and budgeting’ and ‘Managing medical practice’ among physicians, all inter-scale correlations were <0.70 (range 0.43–0.61). Under testing for construct validity, the subscales were positively correlated with ‘formal management roles’ of physicians and nurses.

**Conclusions:**

The professional involvement scales appear to yield reliable and valid data in European hospital settings, but the scale ‘Managing medical practice’ for nurses needs further exploration. The measurement instrument can be used for international research on clinical management.

## Introduction

Drawing clinicians into management practice is increasingly seen as an effective lever for improving the management of hospitals, and ultimately hospital performance [1–5]. Previous research in this area demonstrates that hospital organizations are complex and difficult to manage [6] and that hospital management needs to be improved [7, 8], especially at a time of increased scrutiny of hospital performance as reflected in tighter financial controls, increased regulations and competition [9–11].

Traditionally, hospital organizations have been conceptualized as dual organizations or so-called professional bureaucracies [12]. This concept highlights the two, often competing, organizational logics that inform the organization and management of care delivered in hospital settings [13–15]. In short, clinicians exercise a discretionary role in decisions about individual patients' treatment that inevitably affect overall resource allocation, whereas hospital managers are concerned with treating the needs of a whole population in a context of finite resources. In this perspective, it is understandable that relations between managers and clinicians are often fraught with difficulty and therefore can serve to impede the effective management of hospitals.

Despite international interest in clinical management, the evidence base to inform policy and practice in this area is weak. Most research focuses on the so-called hybridization of clinical and management roles, examining how clinicians cope with managerial duties, and how these dual roles change the nature of professions [4, 16, 17]. Although there is knowledge about how management roles have been introduced and taken up by clinicians in the various hospital systems per country, the overall picture explaining different pathways of change remains limited, especially with regard to how (if at all) clinical management makes a difference to the quality of care delivered [1, 2, 18]. Thus, while there is an international trend towards clinical management [19], we currently know very little about how such processes arise in different hospital systems. Even less clear is how one might explain variations in the practice of clinical management and how clinical managers may influence clinical management and hospital performance.

In this light, comparative, longitudinal and cross-sectional research would be timely and be a welcome addition to the existing body of evidence that mainly comprises of studies that are national and qualitative in nature [10, 19]. We therefore developed and validated a 21-item scale for measuring the professional involvement of leading physicians and nurses in European hospital management as one dimension of clinical management. Hereby, we defined professional involvement as ‘the level of influence of leading physicians and nurses in hospital management decision-making areas’. Specifically, the aim of this paper is 2-fold: (i) to develop and (ii) to validate an instrument for measuring professional involvement among physicians and nurses across seven European countries.

## Methods

### Participants

Our study was part of a European research project called DUQuE, which aimed at deepening the understanding of quality improvement in European hospitals [20]. The DUQuE project was a multi-method, cross-sectional study, which collected quality-related information from European hospitals between May 2011 and February 2012. Seven countries were included: Czech Republic, France, Germany, Poland, Portugal, Spain and Turkey. In each country, 30 hospitals were randomly recruited, provided that they had >130 beds and treated patients with acute myocardial infarction, hip fracture, stroke and deliveries [21]. Within each hospital, we asked 30 leading clinicians (15 physicians and 15 nurses) to answer questions about their professional involvement in hospital management. With the term ‘leading’, we broadly referred to those clinicians who had a leading role within the hospital, and who may also fulfil a formal management role. This role implies leading any number of employees. By using this broader inclusion criterion, we hoped to avoid the self-exclusion of leading clinicians whose hospitals did not have formalized clinical management structures and roles. For our study, 6300 leading physicians and nurses were eligible to be included in our study.

### Measurement instrument for professional involvement

Although clinical management is a broad concept that includes clinical management structures and the execution of formal management roles [19], we assumed that focusing on the level of influence on hospital management decision-making, i.e. professional involvement, would be less context-specific, and, thus, more appropriate for international research. That is, in every hospital system, clinicians influence management decisions whether or not their influence is formalized through formal management roles. Even so, we assumed that the outcome of clinical management in terms of the levels of influence on decision-making would be more relevant than the clinical management structure and processes that pertain to fulfilling clinical management roles.

There were no standardized and validated questionnaires available for measuring professional involvement within hospitals in an international comparative research project—the available empirical studies were mostly country specific and qualitative in nature. Therefore, we decided to develop a professional involvement scale ourselves, partly building on the questionnaire developed by Kruijthof in the Netherlands [22]. The latter questionnaire was used in a national survey among a representative sample of medical specialists in the Netherlands in 2003, focusing on how medical specialists felt about their role and position within the hospital organization. One part of this questionnaire focused on measuring the actual and desired participation in hospital management decision-making. We adapted the relevant items from this questionnaire to fit our research purposes and the international context by rephrasing the wording and by adding items based upon expert opinion. Furthermore, we adapted the questionnaire so that it could be completed by nurses, as nursing professionals were included in the DUQuE project.

Adjustments resulted in a 21-item questionnaire, where each item represented a unique hospital management decision-making area (Appendix). For example, one item asked how respondents participate in the decision-making process on managing the budget of an inpatient unit; another on dealing with the poor performance of colleagues. Respondents could answer on a four-point scale with the following options: 1 = No involvement, 2 = Giving an opinion, 3 = Shared decision-making, 4 = Final decision-making responsibility. We opted for these answering categories as we considered the four answer options more precise and easier to interpret than a Likert-scale using categories ranging from fully disagree to fully agree.

The original English questionnaire went through a process of forward–backward translation and piloting [23]. As a consequence, the questionnaire was available in eight languages: Czech, English, German, French, Polish, Portuguese, Spanish and Turkish. For practical reasons, we did not validate the questionnaire for each language separately, but analysed data of all versions/languages altogether.

### Data collection

The leading physicians and nurses were identified by the hospital coordinators responsible for the data collection within that hospital. Respondents were invited by a letter to participate and personally by the DUQuE-project country coordinator. Questionnaires were completed anonymously and directly entered in the online data platform.

### Data analyses

First, data cleaning was performed to prepare for the statistical analyses. After describing the study sample using appropriate statistics, we investigated the factor structure of the questionnaire for nurses and physicians separately using split file principal components analysis with varimax rotation. We retained factors of subscales with an eigenvalue of at least 1 and three or more item loadings [24, 25]. Individual items were assigned to the subscale on which they had the highest factor loading, with a minimum acceptable loading being 0.3 (i.e. any item that did not load with at least 0.3 was excluded for scale optimization). If an item loaded equally well on two subscales, face validity was used to choose the final destination subscale. Any respondent missing questions for three or more contributing questions was not included in the construct definition sample (i.e. for the factor analysis and reliability testing). Any respondent missing questions for any particular subscale was excluded from analysis involving that subscale.

We examined internal consistency reliability using Cronbach's alpha, with an alpha of at least 0.70 seen as acceptable [26, 27]. Homogeneity of each subscale was verified by item-total correlation (corrected for item overlap), which had to be >0.40 to be acceptable. We assessed the degree of overlap between the subscales using Pearson's correlation coefficient, such that a correlation coefficient of <0.70 was seen as evidence of non-redundant subscales [28, 29]. Finally, we evaluated the external validity of the construct by performing pairwise correlations between the construct subscales and several questions pertaining to the respondent's formal management role, for physicians and nurses separately. All statistical analyses were carried out in SAS (version 9.3, SAS Institute, Inc., NC, 2001).

## Results

### Sample characteristics

There were 188 hospitals participating in the study (Table 1). Most hospitals were public and medium-sized with 200–500 beds, and almost half of the hospitals had a teaching profile. There were 1670 leading physicians and 1716 leading nurses who completed the questionnaire, yielding an overall response rate of 59 and 61%, respectively, for the 188 hospitals included in our study. Questionnaire completeness was high; 1505 physicians (90%) and 1483 nurses (86.4%) responded to all questions in the survey and only 111 (6.6%) physicians and 219 (12.7%) nurses missed responses that were required to calculate two or more of the subscales. The latter two groups were excluded from analyses.

**Table 1:** Characteristics of the study sample

Characteristics of the hospitals	*N* (%)		
All hospitals	188 (100)		
Teaching hospitals	81 (43.0)		
Public hospitals	156 (82.9)		
Approximate number of beds in hospital			
<200	18 (9.5)		
200–500	79 (42.0)		
501–1000	62 (32.9)		
>1000	29 (15.4)		
Characteristics of the respondents	All^a^	Physicians^b^	Nurses
Total number of respondents, *N* (%)	3386 (100)	1670 (49.3)	1716 (50.6)
Gender, *N* (%)			
Male	1388 (40.9)	1151 (68.9)	237 (13.8)
Female	1978 (58.4)	510 (30.5)	1468 (85.5)
Gender missing	20 (0.5)	9 (0.5)	11 (0.6)
Age (years), mean (SD)	47.3 (8.5)	49.3 (8.3)	45.4 (8.2)
Number of years since completion of professional training, mean (SD)	21.9 (9.7)	21.6 (9.9)	22.2 (9.6)
0–5 years, *N* (%)	196 (5.7)	106 (6.3)	90 (5.2)
6–10 years, *N* (%)	277 (8.1)	139 (8.3)	138 (8.0)
11–20 years, *N* (%)	928 (27.4)	483 (28.9)	445 (25.9)
21+ years, *N* (%)	1847 (54.5)	914 (54.7)	933 (54.3)
Missing, *N* (%)	138 (4.0)	28 (1.6)	110 (6.4)
Member of professional society, *N* (%)	2339 (69.0)	1464 (87.6)	875 (50.9)
Formal management role in the hospital, *N* (%)			
No formal management role	257 (7.5)	165 (9.8)	92 (5.3)
Formal management role at the department level only	1801 (53.1)	864 (51.7)	937 (54.6)
Formal management role at the hospital level only	256 (7.5)	90 (5.3)	166 (9.6)
Formal management role at both the department and hospital level	557 (16.4)	293 (17.5)	264 (15.3)
Formal management role missing/unknown	515 (15.2)	258 (15.4)	257 (14.9)

^a^Excluding professionals who are missing responses for >2 of 4 professional involvement subscales.

^b^Including attending physicians and residents-in-training.

### Reliability and validity of the professional involvement scale

The principal components analysis yielded two versions of the professional involvement scale, one for leading physician respondents and one for the leading nurse respondents (Table 2). The major difference is that physician responses to Q1 and Q3 will be considered regarding recruitment and dismissal whereas the corresponding items Q2 and Q4 will be considered for nurses regarding recruitment and dismissal. Since we expect that nurses will have little involvement in recruitment and dismissal of physicians, Q1 and Q3 are fully excluded for nurse respondents. Q2 and Q4 for physician respondents moved to a physician-only construct entitled managing nursing practice. The labelling of the subscales was done on theoretical grounds whereby a term was chosen best fitting the grouped hospital decision-making areas.

**Table 2:** Item and scale characteristics, internal consistency and item-total correlations

Item no.	Scale and items	Factor loadings on primary scale		Internal consistency: Cronbach's alpha		Corrected item-total correlations	
		Physicians	Nurses	Physicians	Nurses	Physicians	Nurses
‘How would you describe your participation within the following decision-making areas?’ 1 = ‘No involvement’, 2 = ‘Giving an opinion’, 3 = ‘Shared decision-making’, 4 = ‘Final decision-making responsibility’							
	Administration and budgeting (*N* = 1626^a^/1642^b^)			0.863	0.814		
Q1	Recruitment and selection of medical specialists^c^	0.775	–			0.701	–
Q2	Recruitment and selection of nurses^d^	–	0.792			–	0.686
Q3	Dismissal of medical specialists^c^	0.776	–			0.697	–
Q4	Dismissal of nurses^d^	–	0.778			–	0.677
Q5	Dealing with poor performance of colleagues	0.759	0.415			0.699	0.370
Q6	Managing budget of inpatient unit	0.722	0.660			0.674	0.604
Q7	Managing hospital admissions	0.600	0.521			0.561	0.475
Q8	The decoration of waiting rooms	0.485	0.514			0.455	0.465
Q9	Human resource management	0.684	0.657			0.648	0.604
	Managing medical practice (*N* = 1648^a^/1584^b^)			0.743	0.607		
Q10	Organization of medical training	0.642	0.510			0.546	0.392
Q11	The content of protocols for medical treatment and diagnosis	0.665	0.564			0.573	0.445
Q12	A new multidisciplinary consult	0.655	0.460			0.570	0.356
Q13	Medical collaboration with primary care (general practitioners, dentists, pharmacists, etc.)	0.536	0.460			0.459	0.357
	Strategic management (*N* = 1609^a^/1625^b^)			0.813	0.782		
Q14	Allocation of hospital beds to departments	0.624	0.513			0.563	0.447
Q15	Allocation of hospital budget	0.725	0.679			0.660	0.608
Q16	Allocation of operating theatre time to specialties	0.454	0.567			0.410	0.498
Q17	Long-term strategic planning	0.710	0.603			0.632	0.518
Q18	The reorganization of the hospital	0.747	0.680			0.663	0.597
Q19	Setting the price and/or volume of physician services	0.591	0.589			0.532	0.515
	Managing nursing practice (physician respondents only)(*N* = 1601^a^)			0.804	–		
Q20	Organization of nursing training	0.668	–			0.606	–
Q21	The content of protocols for nursing care and diagnosis	0.628	–			0.564	–
Q2	Recruitment and selection of nurses	0.790	–			0.684	–
Q3	Dismissal of nurses	0.741	–			0.622	–

^a^Sample size for physicians, excluding professionals who are missing responses for >2 of 4 professional involvement subscales.

^b^Sample size for nurses, excluding professionals who are missing responses for >2 of 4 professional involvement subscales.

^c^Included in the physicians scale but not the nurses scale.

^d^Included in the nurses scale but not the physicians scale.

Cronbach's alpha as a measure of the internal consistency was good for the physicians' professional involvement, ranging from 0.74 for the subscale ‘Managing medical practice’ to 0.86 for the subscale ‘Administration and budgeting’. Cronbach's alpha was lower for the nurses' professional involvement, ranging from 0.61 for ‘Managing medical practice’ to 0.81 for ‘Administration and budgeting’. All subscales except the ‘Managing medical practice’ subscale for nurses achieved reliability coefficients of >0.70.

The item-total scale correlations were acceptable within the range of 0.41 to 0.71 for physician respondents, and the range of 0.36 and 0.69 for nurse respondents. All items of a scale scored within this range. The scores for the subscales for nurses were consistently lower than those for physicians.

The inter-scale correlation matrix (Table 3) ranged from 0.43 between ‘Strategic management’ and ‘Managing nursing practice’ for physicians and between ‘Strategic management’ and ‘Managing medical practice’ for nurses to 0.74 between ‘Administration and budgeting’ and ‘Managing medical practice’ for physicians. For two subscales, the inter-scale correlation was >0.70, the criterion for non-redundancy.

**Table 3:** Inter-(sub)scale correlations for physicians and nurses separately

	Professional involvement			
	Administration and budgeting	Managing medical practice	Strategic management	Managing nursing practice
Physicians				
Administration and budgeting	1			
Managing medical practice	0.74	1		
Strategic management	0.61	0.45	1	
Managing nursing practice	0.50	0.44	0.43	1
Nurses				
Administration and budgeting	1			
Managing medical practice	0.50	1		
Strategic management	0.54	0.43	1	-

Table 4 displays the Pearson correlations of each of the subscales with the questions on ‘formal management roles’. The correlation coefficients were sizeable when comparing the subscales ‘Administration and budgeting’, ‘Managing medical practice’ and ‘Strategic management’ with formal management role questions for physicians. For instance, the question pertaining to management of physicians and nurses was very highly correlated with ‘Administration and budgeting’ (*r* = 0.57) and ‘Managing medical practice’ (*r* = 0.56) subscales. Correlations for the ‘Managing nursing practice’ subscale or for the Nurses subscales were not as remarkable.

**Table 4:** Professional involvement scale validation: pairwise correlations^a^ with ‘formal management role’ questions

Professional involvement subscales	Formal management role 1 = No formal management role, 2 = formal management role at department or hospital level, 3 = formal management role at both department and hospital level	Q2–Q4: ‘Please indicate how you personally fulfil your management role’ 1 = Strongly disagree, 2 = Somewhat disagree, 3 = Somewhat agree, 4 = Strongly agree		
		Q2: I operate as an intermediary between physicians/nurses and the hospital management	Q3: I shape the conditions for medical/nursing practice at unit level	Q4: I manage performance of physicians/nurses
Physicians				
Administration and budgeting	0.25	0.47	0.41	0.57
Managing medical practice	0.28	0.47	0.47	0.56
Strategic management	0.30	0.32	0.26	0.29
Managing nursing practice	0.15	0.23	0.22	0.29
Nurses				
Administration and budgeting	0.12	0.18	0.24	0.31
Managing medical practice	0.15	0.10	0.17	0.16
Strategic management	0.22	0.08	0.07	0.08

^a^Pearsons correlation coefficients.

## Discussion

### Main findings

This study demonstrates that the two professional involvement scales appear reliable and valid in measuring professional involvement of leading physicians and nurses in hospital management at a pan-European level. Based upon the DUQuE sample, the psychometric properties of both professional involvement scales proved to be sufficient, except for the inter-correlation score of two subscales within the physician scale.

Clinical management is on the rise in European hospital systems [1, 5, 19]. This study developed professional involvement scales that can be used for the systematic evaluation of leading physicians' and nurses' decision-making responsibilities in hospital management, and in clinical management more generally. Our findings provide empirical support for the reliability and validity of the results obtained from the scales completed by leading physicians and nurses working in hospitals across the seven European countries. The investigation of the psychometric qualities indicates that both scales can be used to measure and compare the professional involvement of leading physicians and nurses with their hospitals located in the seven European countries.

The results of the factor analysis indicate that the professional involvement scale yields four relevant areas of professional involvement of leading physicians and three of them were also relevant for leading nurses. We note, however, that the high inter-correlation score between the ‘Administration and budgeting’ and the ‘Managing medical practice’ subscales within the physician scale could indicate that some items within those scales might provide some redundant information. The various decision-making areas in hospital management might not be mutually exclusive.

The modest correlations of each of the subscales with the questions on formal management roles provide an intuitive support for the professional involvement scales being part of the phenomenon of clinical management. This explanation is premised on the assumption that clinicians who take up a formal management role will be more shared or final decision-makers in hospital management.

As such, both professional involvement scales provide sufficient basis for further international research in the area of leading physicians and nurses in hospital management. Given the limited international comparative research on clinical management, we believe that the quantitative results obtained in our study are important. The results offer a shared starting point for discussing and researching clinical management roles across Europe and thus complement the nation-specific and more qualitative research evidence.

This study offers a novel quantitative profile of the professional involvement of leading physicians and nurses in hospital management and their influence on decision-making in various management areas. We assumed that focusing on professionals' influence on hospital decision-making would be less dependent on nation-specific context since management decision-making is a process common to all hospitals. This focus was also consistent with the overall objective of the DUQuE project, which aimed to test associations between levels of clinical management with the implementation of hospital quality systems. The results of our study, including the high response and the low number of missing values, seem to support this assumption. Moreover, the country coordinators, responsible for both translating the questionnaires and collecting the data, did not report any problems with regard to the professional involvements scales.

Limitations of the DUQuE project are described elsewhere [21], and our study has related limitations. First and foremost, international research on clinical management needs to account for confounding contextual differences across countries [1]. Hospital systems differ significantly in the seven studied European countries. This also influences how clinicians take up managerial roles, and how they actually are involved in hospital management. Research shows that national policies and local organizational factors are often crucial in shaping the level of decentralization of management functions/responsibilities to clinicians [8, 9]. Many observers believe policies across Europe (and beyond) are converging in this area and increasing efforts are being made to strengthen the clinical management within health organizations [1, 18]. However, the evidence supporting this convergence remains weak. While changes in the relationship between management and clinicians have received some attention at national levels, research has lagged behind and few studies in this area have adopted a rigorous, comparative and interdisciplinary perspective [1, 10, 18]. This gap currently forms the rationale of an EU-funded COST Action on ‘Enhancing the role of medicine in the management of European Health systems –implications for control, innovation and user voice’ (see http://www.cost.eu/domains_actions/isch/Actions/IS0903).

A second limitation relates to the contextual differences at country level, including the role of a physician and a nurse within each system; a nurse in France may have a complete other function and status than a nurse in Turkey. This variation has to do with the professionalization of the health care providers over the last 150 years [13, 28]. These processes have resulted in different levels of training, registration and prestige among these two professional groups across Europe. As such, our sample of leading physician and nursing respondents might not be completely comparable across the seven countries and introduces bias. In this study, however, the management education and training for physicians and nurses might be more relevant than the medical and nursing training, and professional registration. There is variation across European countries in the availability of management education and training, ranging from post-graduate curricula to in-house trainings or no training at all [18].

Third, the existing body of knowledge about effective clinical management is largely based on medical management and physicians taking up managerial roles in their hospitals. As such, the instrument was primarily developed on the basis of one study that has explored the level of medical management [22]. One sign of this limitation is that findings for the physician subscales were not identical to the nursing subscales. Instead of constructing two completely different scales, we opted to maintain optimal comparability between the scales for leading physicians and nurses and to drop one of the subscales from the nursing scale.

### Implications for research and practice

The professional involvement scales for leading physicians and nurses were initially developed for the purposes of the DUQuE project. Future articles will examine the relationships between professional involvement and the implementation of quality management systems, as well as professional involvement and professional attitudes and behaviours towards quality. However, the scales may be useful in other research projects too. For that purpose, the instruments are available in eight languages: Czech, English, French, German, Polish, Portuguese, Spanish and Turkish. We expect that there will be growing interest in monitoring the levels of clinical management across European hospitals, and especially how levels of clinical management relate to hospital performance. A few research studies already showed associations between high levels of clinical management and hospital performance [2, 29–31].

In addition, at hospital level, the professional involvement scales may be used for management purposes. Hospitals signing up to the idea of clinical management can use both scales to get insight in the self-reported involvement of their leading physicians and nurses in hospital management, and thus how their clinical management policies play out in practice.

## Conclusions

We have described the development and validation of a professional involvement scale for measuring the level of clinical management by physicians and nurses in European hospitals. Our findings suggest that each scale provides reliable and valid data on professional involvement, useful for assessing clinical management across European countries.

## Funding

The study, “Deepening our Understanding of Quality Improvement in Europe (DUQuE)” has received funding from the European Community's Seventh Framework Programme (FP7/2007–2013) under grant agreement n° 241822. Funding to pay the Open Access publication charges for this article was provided by European Community's Seventh Framework Programme (FP7/2007–2013) under grant agreement n° 241822.

## Appendix

[Table MZU014TBA1]

**Table A1 MZU014TBA1:** Instrument
